# Lung Cancer: Are we up to the Challenge?

**DOI:** 10.2174/138920210793175903

**Published:** 2010-11

**Authors:** Luca Esposito, Daniele Conti, Ramyasri Ailavajhala, Nansie Khalil, Antonio Giordano

**Affiliations:** 1 Oncology Research Centre of Mercogliano, Avellino, Italy; 2 Department of Human Pathology and Oncology, University of Siena, Siena, Italy; 3 Human Health Foundation Onlus, Spoleto, Italy; 4 Sbarro Institute for Cancer Research and Molecular Medicine, Center for Biotechnology, Temple University, Philadelphia, USA

**Keywords:** Lung cancer, oncosuppressors, oncogenes, epigenetics of lung cancer, diagnostic tools for lung cancer.

## Abstract

Lung cancer is the leading cause of cancer deaths worldwide among both men and women, with more than 1 million deaths annually. Non–small cell lung cancer (NSCLC) accounts for about 80% of all lung cancers.

Although recent advances have been made in diagnosis and treatment strategies, the prognosis of NSCLC patients is poor and it is basically due to a lack of early diagnostic tools.

However, in the last years genetic and biochemical studies have provided more information about the protein and gene’s mutations involved in lung tumors. Additionally, recent proteomic and microRNA’s approaches have been introduced to help biomarker discovery.

Here we would like to discuss the most recent discoveries in lung cancer pathways, focusing on the genetic and epigenetic factors that play a crucial role in malignant cell proliferation, and how they could be helpful in diagnosis and targeted therapy.

## INTRODUCTION

Lung cancer is the leading cause of cancer-related deaths over the world, among both men and women, with an incidence of over 200000 new cases per year and a very high mortality rate. It is responsible for more deaths than breast, colon and prostate tumors combined [1].

Lung tumors can be divided into two histological groups: non-small cell lung cancer (NSCLC) (80.4%) and small cell lung cancer (SCLC) (16.8%) [2]. NSCLC, consisting mainly of adenocarcinoma, squamous cell and large cell carcinoma, accounts for almost 80% of lung cancer cases, whereas SCLC is slightly more common and all known cases are due to cigarette smoking. 

Many factors potentially contribute to lung cancer formation, e.g. tobacco smoke, ionizing radiation and viral infection, although the mechanisms involved in lung carcinogenesis remain largely unknown.

Lung cancer is often suspected on the basis of abnormal chest imaging and/or non-specific symptoms. Bronchoscopy is generally used as an initial diagnostic tool, consisting of cytopathologic examination of bronchoalveolar lavage, endobronchial brushings and biopsies from the suspect area. Despite this procedure being specific for lung cancer, its sensitivity is low [3]. Therefore, more invasive and expensive diagnostic tests are often required, delaying the diagnosis and the subsequent treatment initiation. Indeed, the main problem of lung cancer disease is a lack of early-diagnosis tools, resulting in more than 60% of patients diagnosed with advanced or metastatic disease [4, 5] and therefore not eligible for a curative surgical resection.

The overall five-year survival rate for patients with NSCLC is less than 15% and has remained largely unchanged for the last three decades. Despite new drugs and therapeutic regimens, surgical resection remains the most promising chance for the ~25% of patients who present early-stage disease (stage I-IIIA), although 65% will relapse within two years [6]. 

These data suggest that strategies for the early detection of lung cancer, aimed at identifying the tumor at a stage in which it is small and locally defined, are urgently needed to increase significantly the chances of a cure [4]. Recent evidence shows that low dose spiral computed tomography (CT) detects lung cancer at smaller sizes and earlier than chest X-ray (CXR) that failed to identify 79% of lung cancers that were smaller than 2 cm [7].

Another major problem linked to lung cancer disease, as for other cancers, is the need to find new and more tailored chemotherapeutical.

The variable response to chemotherapy with platinum-based regimens in NSCLC is well recognized clinically. Patients crossing from one regimen to another in clinical trials commonly show responses [8], suggesting that it might be possible to optimize the therapy for individual patients once has been determined which regimen would be most effective. 

In this short review we summarize what are the main genetic alterations involved in lung cancer disease and what is the prospective for the future development of new biomarker and diagnostic tools to improve the early detection of lung cancer. 

## GENETIC AND EPIGENETIC ALTERATIONS IN LUNG CANCER

Different factors contribute to lung cancer formation: tobacco smoke, ionizing radiation and viral infection are among the most well established. However, the mechanisms involved in lung carcinogenesis remain largely unknown to date [9].

As most other cancers, several genes are involved in lung cancer disease [9], which is initiated by the activation of oncogenes or inactivation of tumor suppressor genes [10].

The proto-oncogene *KRAS* is often mutated and is responsible for 10–30% of lung adenocarcinomas [11, 12].

*	MYC* and Cyclin D1 [13] are amplified and over-expressed in 2.5–10% and 5% of NSCLC, respectively.

ERBB2 (also known as HER-2/neu) or BCL2 over-expression are involved in 25% of cases [14]. Farther, many studies have found ERBB2 mutations (exons 19-20) in a small subset of patients. These mutations often represent early events in the carcinogenesis of lung adenocarcinoma in never smokers [15, 16]. Novel mutations in *BRAF* were identified through systematic resequencing of oncogenes and are present in about 2% of adenocarcinoma patients and restricted to tumors that did not show *KRAS* mutations [17, 18]. 

The epidermal growth factor receptor (EGFR) that regulates cell proliferation, apoptosis, angiogenesis and tumor invasion [11] is over-expressed or in certain cases affected by oncogenic mutations in NSCLC and is one of the major target for lung cancer therapy. Furthermore, some activating mutations in EGFR, mainly deletion mutations in exon 19 and the single L858R point mutation in exon 21, are associated with increased response and survival after tyrosine kinase inhibitor therapy, whereas the T790M point mutation or insertion mutations in exon 20 of EGFR, are associated with failure to respond [19-22]. 

Basically, mutations involving EGFR, ERBB2 and KRAS are mutually exclusive and are thought to represent early events in the carcinogenesis of lung adenocarcinoma in never (EGFR and ERBB2) and current smokers (KRAS) [23].

Other oncogenes whose expression has been found altered in lung cancer, include *MET*, *NKX2-1*, and *PIK3CA* [11]. 

The tyrosine kinase protein, SRC is also overexpressed and activated in epithelial tumors, and the levels of expression or activation generally correlate with disease progression [24], although activating mutations are rare [25]. Studies have shown that SRC is activated in NSCLC tumor tissues [26, 27], and its inhibition leads to decreased anchorage-dependent cell growth and to cell cycle arrest and apoptosis [28, 29]. 

Recently, the fusion of the anaplastic lymphoma kinase (*ALK*) with the echinoderm microtubule-associated protein-like 4 (*EML4*) has been identified in a subset of NSCLC. Approximately 5% of all NSCLC cases contain an *EML4-ALK* translocation. It occurs in mutual exclusion to *EGFR* and *KRAS* mutations and is associated with nonsmokers. Since *ALK* tyrosine kinase activity is oncogenic both *in vitro* and *in vivo*, new *ALK* kinase inhibitors are being evaluated in pre-clinical trials for lung cancer [30, 31].

Also, inactivation of tumor suppressor genes plays an important role in lung carcinogenesis, as for example the tumor suppressor gene *TP53* that is mutated in 60–75% of lung cancer including both NSCLC and SCLC [32]. 

Another important tumor suppressor gene is *LKB1*, whose loss-of-function mutation/deletion is observed in 30% lung adenocarcinomas and 20% of squamous cell carcinomas [33, 34]. 

Recently it has been shown a clearly link between SCR and *LKB1* in NSCLC disease. In particular, SRC and FAK signaling pathways are activated in lung cancers when the tumor suppressor LKB1 is deleted. These findings suggest the use of unique combinatorial therapies for treatment of lung cancers [35].

The role of RB family genes in lung cancer malignancy has been long examined but remains unclear to date.

What is known is that the tumor suppressor RB1 is inactivated in a broad range of human tumors [36, 37], including pediatric retinoblastomas and about 90% of human SCLC. When RB1 is not itself mutated, other alterations in members of the RB pathway are found in human tumors [37-39]. For instance, RB1 is rarely found mutated in lung adenocarcinomas whereas p16^INK4a^, an upstream activator of the RB1 protein and the two related proteins p107 and p130, is frequently inactivated in this tumor type [40].

Recent studies provided genetic evidence that RB1 and p130 have the capacity to act as suppressors of lung adenocarcinoma development, confirming the broad tumor-suppressor potential of the RB family genes and raising the possibility that re-activation or induction of RB family function in lung cancers may be used to slow tumor growth in patients [41]. 

Genetic polymorphisms are also indicated to be involved in lung carcinogenesis, e.g. interleukin-1 [42], cytochrome P450 [43], apoptosis promoters such as caspase-8 [44] and DNA repair proteins such as XRCC1 [45]. 

Epigenetic modifications are now well recognized to significantly contribute to lung cancer tumorigenesis. For example, a great number of aberrantly methylated genes have been identified in lung cancer. A well-studied example is the aberrant promoter methylation of the tumor suppressor gene p16 which leads to gene silencing, an early event in tumorigenesis [46-48]. Additional examples include H-cadherin [49], death-associated protein kinase 1 (DAPK1) [50], 14-3-3 sigma [51] and the candidate tumor suppressor gene RASSF1A [52].

Although research based on known genes, proteins and epigenetic alteration has already yielded new information, during the past decade the microRNA (miRNA) research-field has been extensively studied and it may also lend insights into the biology of lung cancer, as well as for cancer in general.

MiRNAs are small non-coding RNAs about 22 nt long that play key roles in gene expression regulation by modulating the translation and degradation of target mRNAs through base pairing to partially complementary sites [53-55].

MmiRNA microarray analysis for lung cancer were recently examined to investigate miRNA involvement in lung carcinogenesis and the results obtained show that miRNAs could discriminate lung cancers from healthy lung tissues, suggesting that miRNA expression profiles could be diagnostic and prognostic markers of lung cancer [56] and also allow for the differential diagnosis between lung adenocarcinoma and mesothelioma [57].

Recent studies have reported some examples of miRNAs implication in lung cancer. For example miR-29 family members directly target both DNA methyltransferases *DNMT 3A* and -*3B*. In particular, it has been shown that expression of miR-29 family members is inversely correlated with DNMT3A and -3B expression in lung cancers and that these miRNAs down-modulate expression levels of both enzymes. Furthermore, enforced expressionof these miRNAs in lung cancer cells leads to reduced global DNA methylation, restores expression of TSGs, and inhibits tumorigenicity both *in vitro* and *in vivo* [58].

Another important report shows that miR-107 and miR-185 are down regulated in lung cancer compared with normal lung and their reintroduction in NSCLC cell lines is able to suppress cell growth [59]. Similarly, miR-15a/miR-16, which induce RB1-mediated cell cycle arrest by the down-regulation of G1 cyclins are downregulated in NSCLC [60].

Farthermore, we must remember the role of telomere and telomerases that recently have been demonstrated to be involved in lung cancer.

Telomeres are nucleoprotein complexes located at the end of eukaryotic chromosomes, which role is to prevent them from degradation, end to-end fusion and rearrangement.

Recently, telomere length has been proposed as prognostic factor in NSCLC, reflecting indirectly chromosomal instability.

Telomerase and telomeric complex play a key role in lung tumor progression. As telomere maintenance is essential to tumor cell proliferation, several approaches have been developed to target either telomerase or telomeric complex. Anti telomerase strategies can either target hTERT or hTERC, in addition to modulation of telomerase regulators at the transcriptional and post-transcriptional levels. More recently, the use of specific ligands leading to G quadruplex telomeric structure stabilization and therefore to limitation of telomerase accessibility to its target appears as a promising area of development [61].

## DIAGNOSTIC TOOLS IN LUNG CANCER

Lung cancer is the most prominent cause of cancer-related mortality worldwide. About 60% of those diagnosed with lung cancer die within one year after diagnosis and the five-year survival for all patients with lung cancer is only 16%, a percentage that has not improved significantly in the past 10 years [1]. Although many insights into the molecular pathology of lung tumors have been achieved, additional information is critical for the development of targeted treatments and of early diagnostic methods. Diagnosis and accurate staging of lung cancer is essential for selection of appropriate curative or palliative therapy and affects patient prognosis. Both invasive and non-invasive procedures are used for this purpose.

Thorax computerized tomography (CT) and positron emission tomography (PET) are non-invasive techniques used to detect lymph node involvement and histological sampling of lymph nodes, but often these techniques are not sufficient and thus invasive techniques such as transbronchial needle aspiration (TBNA), needle aspiration by endobronchial ultrasound (EBUS) and mediastinoscopy are frequently used to recover histological samples from lymph nodes [62]. Since patients often present poor general conditions or severe hypoxemia due to coexisting diseases (COPD, heart failure, etc.), it may not be possible to use invasive procedures for diagnosis and staging in some of the patients with lung cancer.

Recent studies provided first evidence for the potential usefulness of a blood-based test for lung cancer diagnosis [63].

For example the mutational status of EGFR can be readily detected in primary tumors and the correlation between EGFR mutations and EGFR TKI (Tyrosine Kinase Inhibitors) sensitivity has been validated in several clinical trials, but it may be difficult to obtain tumor tissues for such analysis. Therefore, since plasma samples of patients with NSCLC often contain circulating DNA derived from tumor tissues, plasma samples have been used for detecting genetic alterations, in particular for EGFR [64]. 

The research of new biomarkers is important not only for improving diagnosis but also it may offer promise in optimizing treatment. Understanding the genetic mechanisms affecting drug activity and response to treatment is a major challenge for establishing an individualized chemotherapy. 

To date, cisplatin and platinum-containing drugs are routinely used for treatment of NSCLC and are known to have vital role.

DNA adducts, due to cisplatin treatment, are mainly repaired by nucleotide excision repair pathway and proteins such as excision repair cross complementing 1 (ERCC1), ribonucleotide reductase subunit M1 (RRM1) and breast cancer susceptibility gene 1 (BRCA1) have an important role in this process. 

Recently, ERCC1, RRM1 and BRCA1 and TS (Thymidylate synthase) [65] have been confirmed as predictive markers of treatment response and survival benefit in patients with lung cancer and prospective studies investigating the efficacy of their determination in larger set of patients are currently ongoing [66].

But to date more than two thirds of lung cancer tumors are diagnosed at late stages when the survival rate is low [4] and thus the main purpose and also the major obstacle of lung cancer research therapy is to detect lung cancer at an early stage and when it is still locally defined.

## CONCLUSIONS

Lung cancer is the leading cause of death over the world and the only chance of cure for patients affected from this kind of cancer is surgical resection.

This is mainly due to the fact that several factors are involved in lung cancer develoment and progression and to date the diagnostic methods available for an early and efficient detection are not sufficient.

Although lung cancer research data have accumulated dramatically during the past several years, there is no database specifically focusing on lung cancer molecular biology available yet.

In this short review we have summarized some of the factors contributing to lung cancer, but it is just an overview of the most important proteins involved in lung cancer disease, which are often mutated or present an unusual pattern of expression compared to the healthy tissue.

All together these data are important for understanding the nature behind this type of cancer and also to understand that basic research on proteins, miRNAs and all the other epigenetic modifications could be used to develop more powerful diagnostic tools, as well as prognostic or predictive markers or even for the development of new targeting therapies for lung cancer.
